# Invariant NKT Cells Drive Hepatic Cytokinic Microenvironment Favoring Efficient Granuloma Formation and Early Control of *Leishmania donovani* Infection

**DOI:** 10.1371/journal.pone.0033413

**Published:** 2012-03-22

**Authors:** Florence Robert-Gangneux, Anne-Sophie Drogoul, Octavie Rostan, Claire Piquet-Pellorce, Jérome Cayon, Mariette Lisbonne, André Herbelin, Hugues Gascan, Claude Guiguen, Michel Samson, Jean-Pierre Gangneux

**Affiliations:** 1 Centre Hospitalier Universitaire de Rennes, Laboratoire de Parasitologie, Rennes, France; 2 INSERM U1085, IRSET (Institut de Recherche en Santé Environnement Travail), Université Rennes 1, Rennes, France; 3 INSERM U564, Université d’Angers, Angers, France; 4 INSERM U935, Université Paris-Sud 11, Villejuif, France; Agency for Science, Technology and Research - Singapore Immunology Network, Singapore

## Abstract

The development of inflammatory granulomas around infected Kupffer cells is necessary for hepatic parasite clearance during visceral leishmaniasis. Invariant NKT (iNKT) cells are predominant T cells in the mouse liver and can synthesize large quantities of IL-4 and IFN-γ, two cytokines involved in granuloma formation. This study analyzed the role of iNKT cells in the hepatic immune response during *Leishmania donovani* infection, using a murine model of wild-type (WT) and iNKT cell-deficient (Jα18^-/-^) C57BL/6 mice sacrificed 15, 30 or 60 days post-infection. We recorded hepatic parasite loads, cytokine expression, and analyzed granulomatous response by immunohistochemistry and hepatic immune cell infiltration by flow cytometry. Whereas WT animals rapidly controlled the infection and developed an inflammatory response associated with a massive influx of iNKT cells observed by flow cytometry, Jα18^-/-^ mice had significantly higher parasitic loads on all time points. This lack of control of parasite burden was associated with a delay in granuloma maturation (28.1% of large granulomas at day 60 versus 50.7% in WT). Cytokine transcriptome analysis showed that mRNA of 90/101 genes encoding chemokines, cytokines and their receptors, was underexpressed in Jα18^-/-^ mice. Detection of IL-4 and TNF-α by ELISA in liver extracts was also significantly lower in Jα18^-/-^ mice. Consistent with flow cytometry analysis, cytokinome profile in WT mice showed a bias of expression towards T cell-chemoattractant chemokines on D15, and displayed a switch towards expression of granulocytes and/or monocytes -chemoattractant chemokines on D60. In Jα18^-/-^ mice, the significantly lower expression of CXCL5, MIP-2 and CCL2 mRNA was correlated with a defect in myeloperoxidase positive-cell attraction observed by immunohistochemistry and with a lower granulocyte and monocyte infiltration in the liver, as shown by flow cytometry. These data indicate that iNKT cells play a role in early and sustained pro-inflammatory cytokine response warranting efficient organization of hepatic granulomas and parasite clearance.

## Introduction

The liver is a target organ in a number of infectious diseases and its own particular way of both tolerating antigens and clearing blood borne pathogens determines the outcome of infection. During visceral leishmaniasis, the tissue microenvironment influences the control of infection, which is organ-specific [1]. In the liver of mice, experimentally infected with the protozoan parasite *Leishmania donovani*, acquisition of resistance is associated with a hepatic granulomatous response [2], [3]. As in other infections with intracellular microorganisms, granulomas are presumed to represent tissue expression of a successful T cell-dependent immune response [2], [4], [5], leading to attraction of different cell types around infected Kupffer cells [6] and potentialization of their microbicidal activity involving reactive oxygen and nitrogen intermediates [7]. It has been shown that IL-12, IL-4, IFN-γ and TNF-α play a key role in the control of parasite multiplication and liver granuloma formation [8], [9], [10], [11], [12], [13]. Concerning cell effectors, blood monocyte influx is necessary for functional granuloma formation [14] and CD4 and CD8 T cell subsets are crucial for chemokine and cytokine synthesis supporting successful granuloma assembly and function [15], [16], [17]. Polymorphonuclear neutrophils (PMN) have also been shown to play a key role in the early control of hepatic parasite burden in mice [1], [18].

More recently, the role of a peculiar subset of T cells, known as natural killer T (NKT) cells, was described in other models of chronic microbial infection involving granulomatous reactions, such as *Mycobacterium* infection [19], [20], [21]. This unique subset of T cells harbors both NK surface markers such as NK1.1 and αβ T cell receptors capable of recognizing only lipid or glycolipid antigens, either endogenous or exogenous (α-galactosylceramide (α-GalCer)) presented by the MHC class Ib molecule CD1d. Two subtypes of NKT cells can be distinguished: i) invariant NKT (iNKT, or type I) cells, which express a semi-invariant TCR consisting, in mice, of an invariant Vα14Jα18 chain paired with a limited repertoire of β chains mostly biased towards Vβ8, Vβ7 or Vβ2; and ii) the less abundant type II NKT cells, with more diverse TCR expression. In mice, iNKT cells represent a major lymphocyte subtype in the liver [22], [23]. The fact that these cells can be stimulated by glycolipid antigens makes them key candidate effector cells in the early immune response against *Leishmania*, whose main surface components are comprised of glycoinositol phospholipids and lipophosphoglycan (LPG) [24]. It was indeed recently shown that glycosphingophospholipid antigens of *L. donovani* can bind to CD1d [25], and that LPG can activate iNKT cells efficiently [26]. Once activated, iNKT cells rapidly produce large amounts of IFN-γ and IL-4 [27], and various other cytokines promoting either Th-1 or Th-2 immune responses, and modulate a wide range of immune phenomena, whether anti-tumoral, or anti-microbial, or even exacerbating inflammatory responses and tissue damage [28], [29]. Indeed, a consistent body of evidence indicates that iNKT cells may have contrasting roles depending on the initial stimulus and subsequent modulation of other cell types [28], [30], [31], [32], [33]. The iNKT cell subset was shown to play a protective role in cutaneous leishmaniasis in a murine model infected with *L. major* [34], [35], but data are scarce in the field of visceral leishmaniasis. Recently, it has been reported that *L. donovani*-induced expression of signal regulatory protein α on Kupffer cells enhances iNKT cell activation and IFN-γ production in the first day after infection, suggesting early interaction between the parasite and these cells [36]. Amprey *et al*. [26] previously pointed to a role of the whole NKT cell subset in anti-leishmanial protective immunity, using CD1d^-/-^ BALB/c mice, whereas Stanley *et al*. found no improvement of the disease after α-GalCer activation of iNKT cells in mice with previously established or concomitant *L. donovani* infection [33].

In the present study, we investigated selectively the role of the iNKT cell subset, using iNKT cell-deficient C57BL/6 mice (Jα18^-/-^), by examining their potency to contribute or not to a favorable hepatic microenvironment warranting parasite clearance. Using a large scale approach of cytokine transcriptome, coupled to hepatic histology and flow cytometry analysis of liver homogenates, we aimed to finely dissect the role of iNKT cells in the early orientation of the hepatic immune response to *L. donovani* and their long-term effect on the histological response. Our results indicate that iNKT cells participate in the establishment of a sustained cytokine synthesis network, involved in cell attraction within liver granulomas and contribute to enhanced phagocytic functions and parasite clearance.

## Results

### iNKT cells contribute to early control of *L. donovani* burden in the liver

**Figure 1:**
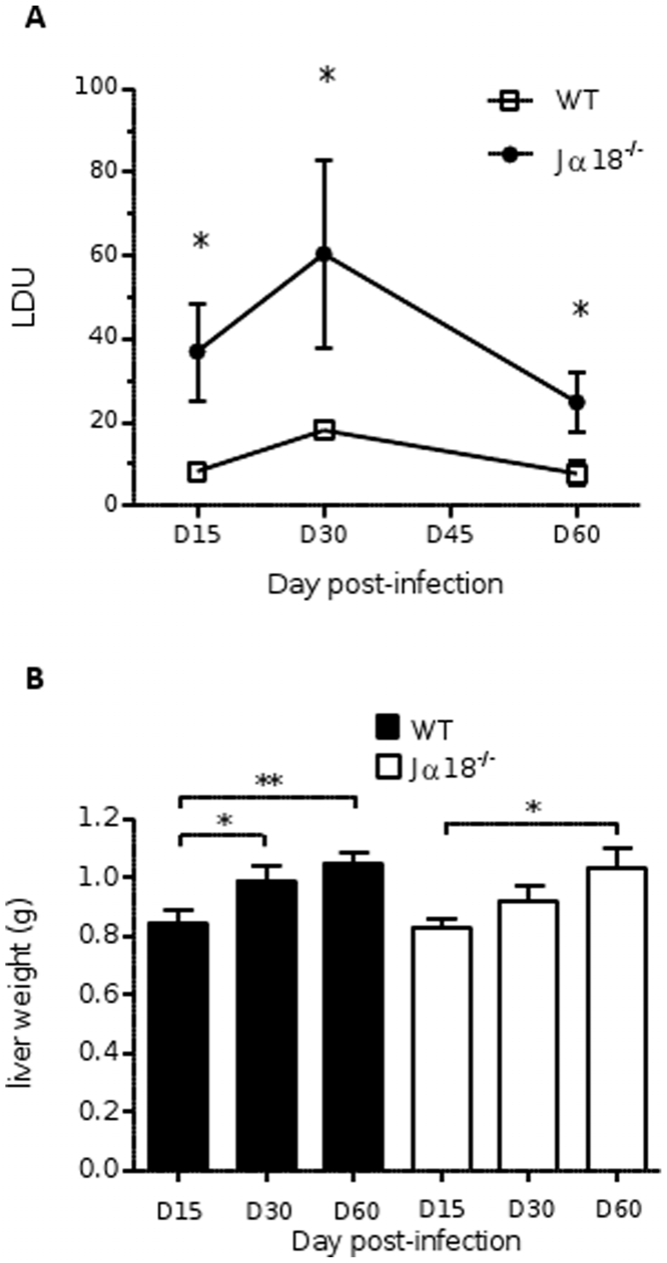
Hepatic parasite burden and liver weight in C57BL/6 WT and Jα18^-/-^ mice after infection with *Leishmania donovani.* (A) Liver parasite burden determined on day 15, day 30, and day 60 post-infection by microscopic counting of Giemsa-stained tissue sections, and expressed as LDU (number of parasites/1000 nuclei × liver weight (mg)). (B) Change in liver weight over the course of infection in WT and Jα18^-/-^ mice. Pooled data from two independent experiments (8–10 mice per group).

Comparison of the course of liver infection between wild-type (WT) and Jα18^-/-^ mice for 60 days after challenge revealed that the parasite burden was higher in iNKT cell-deficient mice at all time points. At D15 post-infection, the liver parasite burden was 4.5-fold higher in Jα18^-/-^ mice (37±11.6 LDU) compared to WT mice (8.3±0.9 LDU, p<0.05) (Figure 1A). At D30 post-infection, the liver parasite burden was still 3-fold higher in Jα18-/-mice (60.5±22.7 LDU vs 18.1±2.3 in WT mice, p<0.01). At D60 post-infection, the hepatic parasite load decreased in both groups, reaching a residual level of 24.9±7.2 LDU and 7.8±2.9, in Jα18^-/-^mice and WT mice, respectively (p<0.05).

Hepato-splenomegaly is a common feature of visceral leishmaniasis. An increase in liver weight was observed in both groups of infected mice, but the weight of the livers obtained from aged-matched WT and Jα18^-/-^ mice did not differ significantly (Figure 1B).

### Jα18^-/-^ mice display an impaired hepatic granulomatous response

**Figure 2:**
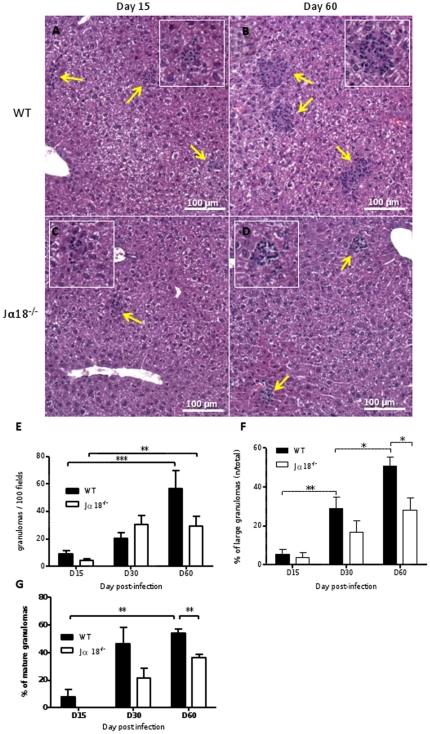
Jα18^-/-^ mice display a qualitatively and quantitatively impaired hepatic granulomatous response after infection with *L.donovani*. Quantification of granuloma formation in the liver of *L. donovani* infected WT and Jα18^-/-^mice by microscopic examination of tissue sections stained with HES, at D15, D30 and D60 post-infection. Representative granuloma foci from WT mice at D15 (A) and D60 (B), and from Jα18^-/-^ mice at D15 (C) and D60 (D) (×100 magnification). (E) Total number of granulomas in 100 microscopic fields (×400) at various time points. (F) Relative percentage of large granulomas (>25 cells) detected in each group of mice. (G) Relative percentage of mature granulomas detected in each group of mice.

In experimental visceral leishmaniasis, resistance to *L. donovani* depends on T cell-mediated formation of tissue granulomas, which are assembled around a core of fused, parasitized resident macrophages. The liver histological response to *Leishmania* infection was quantified by microscopic examination of tissue sections stained with HES (Figure 2A–D). The total number of granulomas in 100 consecutive fields (×400) increased over time post-infection in both groups of mice (p<0.001 in WT) but did not differ quantitatively between the two groups at each time point (Figure 2E). Granulomas were classified into two categories based on their size (i.e. <25 or >25 cells attracted to the foci) and maturation stage, and counted in 100 microscopic fields. The total number of small inflammatory foci was not significantly altered in Jα18^-/-^ mice (not shown). However, when we focused on large-sized (>25 cells) or mature granulomas, that guarantee an effective immune response, it appeared that the mean percentage of large granulomas was almost 2-fold higher in WT compared to Jα18^-/-^ mice, with a significant difference on D60 (50.7±4.8% and 28.1±6%, respectively; p<0.05) (Figure 2F). Similarly, mature well-organized granulomas were more frequently observed in WT mice (54.3±3% versus 36.1±2.6% in Jα18^-/-^ mice at D60; p<0.01) (Figure 2G). In WT mice only, the number of granulomas was negatively correlated with the liver parasite burden (Spearman r = −0.53, p<0.05), suggesting that in WT mice, a stronger granulomatous tissue response was associated with more efficient parasite clearance, whereas in Jα18^-/-^ mice, impaired granuloma maturation could account for enhanced parasite multiplication.

### iNKT cells are recruited early during hepatic *Leishmania* infection in wild-type mice

**Figure 3:**
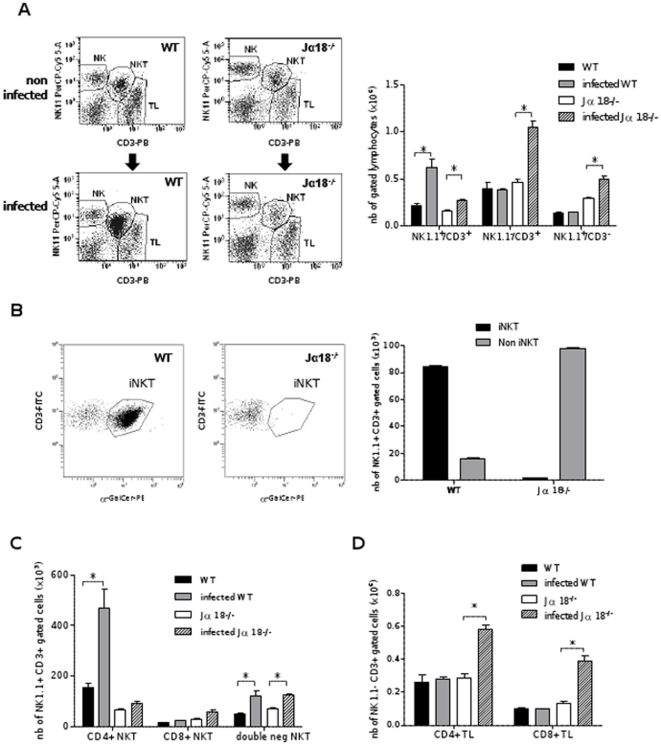
iNKT cells are recruited in the liver of WT mice on D15 after infection with *L.donovani*. Hepatic cell infiltration of C57BL/6 WT and Jα18^-/-^ mice was analyzed by flow cytometry 15 days after infection with *L. donovani*, using an anti-Gr1-FITC, anti-CD11b-PE-Cy7, anti-CD11c-APC, anti-CD4-PB, anti-CD3-FITC, anti-NK1.1-PerCP-Cy-5.5, and anti-CD8-APC-Cy7, and αGalCer-tetramer-PE. (A) Absolute quantification of hepatic NK (NK1.1+/CD3-), NKT (NK1.1+/CD3+), and TL (NK1.1-/CD3+) cell subsets in lymphocyte gated cells.(B) proportion of iNKT and non iNKT cell subsets in each group of mice in CD3+/NK1.1+ gated cells. (C) Absolute quantification of CD4^+^ and CD8^+^ cell subsets in gated NKT cells (CD3+/NK1.1+). (D) Absolute quantification of CD4^+^ and CD8^+^ cells in gated T lymphocytes (CD3+/NK1.1-). 10^6^ cells of liver homogenates were labeled and data were analyzed on 50.000 events. Data are the mean ± SEM of four mice per group.

**Figure 4:**
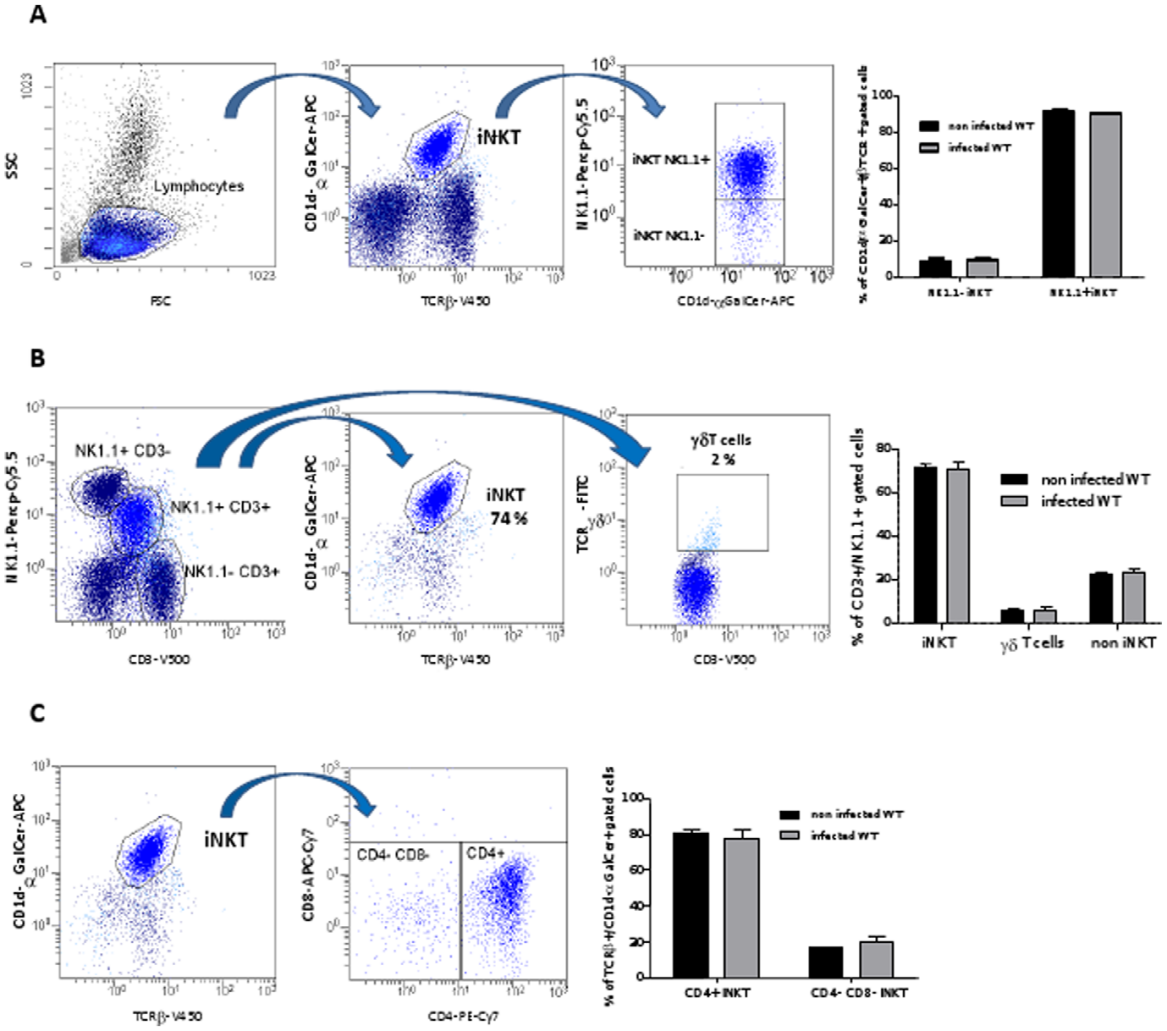
Phenotypic characterization of the iNKT cell infiltrate in the liver of WT mice on day 15 after infection with *L.donovani.* Hepatic cell infiltration of C57BL/6 WT was analyzed by flow cytometry using anti-CD3-V500, anti-NK1.1-PerCP-Cy-5.5, anti-TCRβ-V450, anti TCR γδ-FITC, and αGalCer/CD1d tetramer-APC, anti-CD4-PE-Cy7, anti-CD8-APC-Cy7. (A) Gating strategy allowing to estimate the low percentage of NK1.1- and NK1.1+ cells among TCRβ+/αGalCer/CD1d tetramer+ cells. (B) Gating strategy and evaluation of the percentage of γδ-T cells and non iNKT cells among CD3+/NK1.1+. (C) Percentage of CD4+ and CD4-/CD8- cells among iNKT cells (TCRβ+/αGalCer/CD1d tetramer+). This panel is representative of three independent experiments.

In the aim to understand the cellular factors contributing to a more efficient and earlier parasite control in WT mice, we undertook the quantification of immune cell populations in the liver of WT and Jα18^-/-^ mice 15 days after infection, using flow cytometry. The total amount of NKT cells was quantified using an anti-CD3-PB and an anti-NK1.1-PerCP-Cy-5.5, and showed that this cell subset was more abundant in WT mice than in Jα18-/-mice (13.57±2.6% versus 7.92±0.77, p<0.05), as expected in iNKT-cell deficient mice (Figure 3A). The proportion of iNKT cells among the NKT cell subset was estimated using a tetramer-CD1d/αGalCer-PE antibody which showed that iNKT cells account for 75–85% of the NKT cell subset in WT animals, whether infected or not (Figure 3B). Whereas NKT cells (CD3^+^/ NK1.1^+^ subset) were massively recruited in the liver of WT animals with a nearly 3-fold increase (0.22±0.02 to 0.62±0.09 ×10^6^ cells per liver, p<0.05) 15 days after infection, this cell subset raised only mildly in Jα18^-/-^ mice (0.16±0.01 to 0.27±0.003 ×10^6^ cells per liver). Instead, the lack of iNKT cells in Jα18^-/-^ mice was mainly compensated by T lymphocytes (CD3^+^/ NK1.1^–^ subset) which increased from 0.46±0.02 to 1.14±0.06 ×10^6^ cells per liver, p<0.05) and, to a lesser extent, by NK cells (CD3^–^/NK1.1^+^ subset) which increased from 0.29±0.01 to 0.48±0.03 ×10^6^ cells in Jα18^-/-^ mice (p<0.05) (Figure 3A). The major part of NKT cells in the liver of WT animals were CD4^+^ (p<0.05). Double negative (CD4^–^/CD8^–^) NKT cells was enhanced after infection in both groups of mice (p<0.05, Figure 3C). T cell (NK1.1-/CD3+) infiltration in the liver of Jα18^-/ -^mice was a mix of CD8^+^ and CD4^+^ T cells (p<0.05 for both subsets) (Figure 3D). In the aim to confirm the predominant role of iNKT cells in the hepatic response to infection, a fine characterization of the T cell subsets was undertaken in WT mice, using an 8-coulour antibody panel (Figure 4). It confirmed that iNKT cells (TCRβ+/CD1d-TT+) account for about 80% of CD3+/NK1.1+ cells. Besides, nearly 7% of TCRβ+/CD1d-TT+ are NK1.1-, suggesting that iNKT cell subset is somewhat underestimated when estimating their amount by gating on CD3+/NK1.1+ cells (Figure 4A). Furthermore, using an anti-γδTCR antibody, we could show that the CD3+/NK1.1+ cell subset is only mildly contaminated by γδT cells, since only 2–3% of γδTCR+/CD3+ were found in this gate (Figure 4B). The percentage of these minor subsets, as well as the non invariant NKT cell subset, did not vary after infection. As already shown on the NK1.1+/CD3+ cell subset, iNKT cells were mostly CD4+ (Figure 4C), and the proportion of CD4+ iNKT, and CD4-/CD8- iNKT subsets remained nearly stable after infection. The dramatic increase in the total amount of CD4+ iNKT cells (Figure 3) is explained by the high proportion of these cells (about 80% of all iNKT cells), but as the percentage of all subsets did not vary (Figure 4B), it can be assumed that minor NKT cell subsets were recruited in similar proportion.

Myeloid cells showed slight differences between both genetic backgrounds, but at this stage of infection they represented less than 10% of all hepatic immune cells, and infection did not significantly modify their amount at this time point (data not shown). Taken together, flow cytometry analysis showed that CD4^+^ iNKT cells are strongly attracted in the liver of WT mice, and that the lack of this cell subset is quantitatively compensated in Jα18^-/-^ mice by T lymphocytes and NK cells.

### Jα18^-/-^ mice display an impaired transcriptome of soluble immune effectors

**Figure 5:**
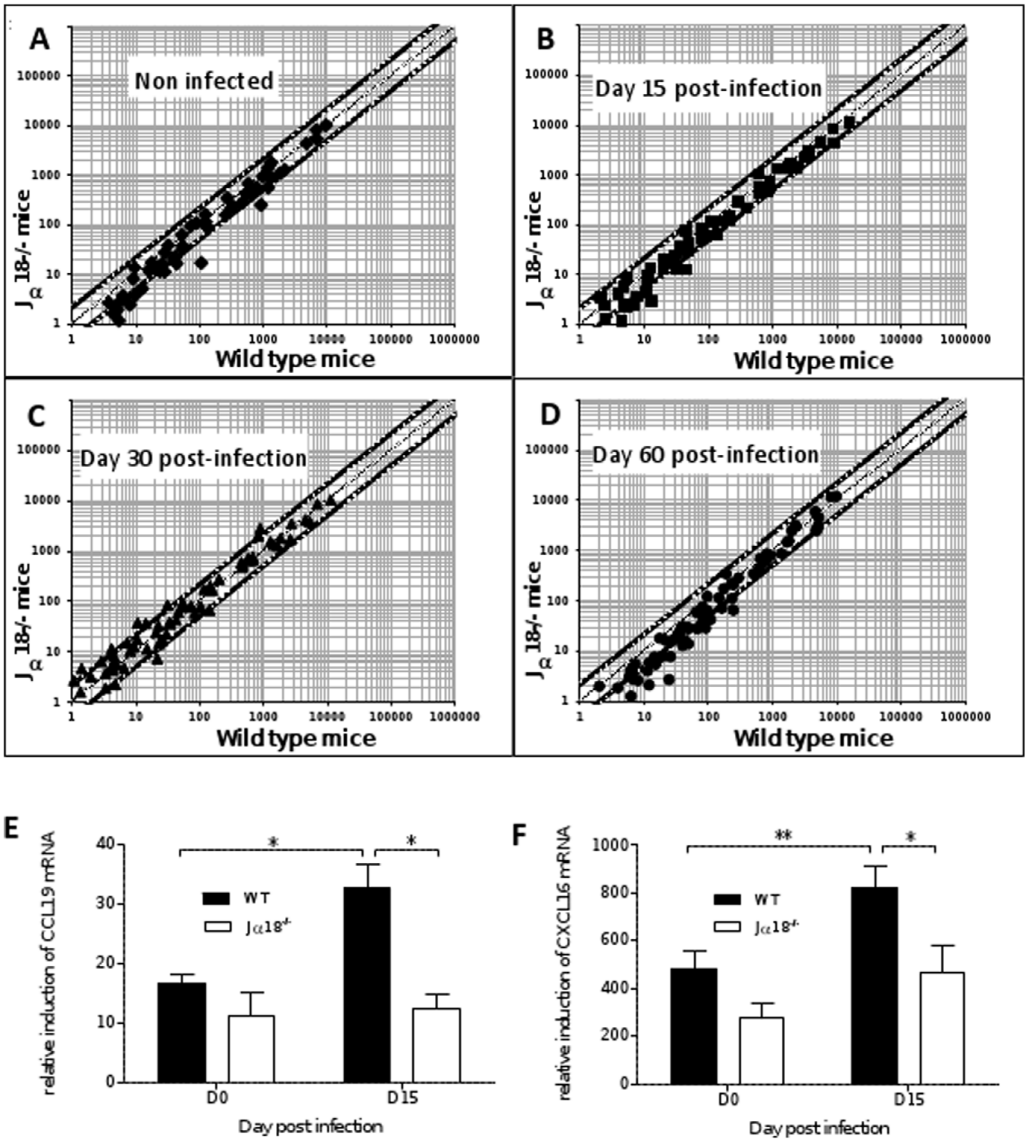
Hepatic transcriptome of cytokines and receptors is altered in Jα18^-/-^ mice. Graphs represent the ratio of genes induction in Jα18^-/-^ mice compared to WT, after normalization on three housekeeping genes, at day 0 (non infected) (A), day 15 (B), day 30 (C) or day 60 (D) after infection, with the central 45° angle line indicating a same level of induction in both backgrounds of mice and the two border lines corresponding to a 2-fold induction in one or the other group of mice. Data are the mean±SEM of triplicate samples pooled from 8 to 10 mice from two independent experiments. Quantification of mRNA of the T cell-chemoattractant chemokines CCL19 (E) and CXCL16 (F) in liver extracts of naïve WT and Jα18^-/-^ mice, 15 days after infection. Data represent mRNA induction normalized on three housekeeping genes, and are the mean±SEM for each group of mice (8–10 mice per group from two independent experiments).

In an attempt to characterize the soluble factors involved in the differential cell recruitment observed in the liver of both groups of mice, we used a high-throughput qPCR approach for quantification of mRNA induction of a large panel of cytokines and chemokines genes (Table S1). Overall, compared to housekeeping genes, high expression levels of genes encoding a number of chemokines and their receptors were observed, whatever the group of mice, probably related to the C57BL/6 background (Figure S1). However, when computing a ratio of relative gene induction between WT and Jα18^-/-^ mice, it revealed that Jα18-/-mice had an overall impaired induction of most cytokines as a result of their individual background (day 0) as well as after infection. Indeed, in Figure 5 most data points (90/101) are located below the curve, indicating a preferential induction in WT animals at a basal level (Figure 5A), as well as after infection with *L. donovani* at any time point (Figure 5B–D). Of these 90 genes, 33 were induced at an at least 2-fold higher rate in WT than in Jα18^-/-^ mice (Figure S2). This stronger gene induction in WT mice probably reflects the deployment of immune effectors participating to anti-parasite defense.

Interestingly, the most significantly under-expressed genes on D15 in Jα18^-/-^ mice encode chemokines or receptors for chemokines involved in activated T cell attraction, i.e. CCL19, CCL27, CXCL16, CCR9, as well as the pan-chemoattractant chemokine CXCL12 and its receptor CXCR4 (Figure S2). Other chemokines were highly induced in both backgrounds (though to a lesser extent in Jα18^-/-^), such as CXCL9, CXCL10, CCL25 and CCL9 and the receptors CCR2, CCR5, CXCR3, CX3CR1 and CXCR6, that could explain the attraction of T cells and NK cells observed by flow cytometry analysis in the liver of Jα18^-/-^ mice (Figure 3). Individual analysis of mRNA induction showed a significant increase of CCL19 and CXCL16 in WT mice at D15 (p<0.05 and p<0.01, respectively) (Figure 5E–F), as well as a higher basal expression level of chemokines capable of attracting iNKT cells, i.e. CXCL9 (3.8-fold), CXCL10 (2.2-fold) and CCL2 (2-fold) (Figure S1), that could explain the early recruitment of iNKT cells observed by flow cytometry at D15. In agreement with this hypothesis, flow cytometry analysis on D15 showed an increase of CXCR3+ cells in WT mice, with a predominant recruitment of CXCR3+ iNKT cells after infection, this lymphocyte cell subset being obviously the main CXCR3+ cell subset in the liver with about 90% of positive cells (Figure S3). Together with iNKT cells though to a far lesser extent, CXCR3-positive T cells and NK cells were also recruited in WT mice (Figure S3), and could partly explain the T and NK cell attraction observed in Jα18^-/-^ mice.

### Pro-inflammatory cytokines are correlated with granuloma formation and are less expressed in Jα18^-/-^ mice

**Figure 6:**
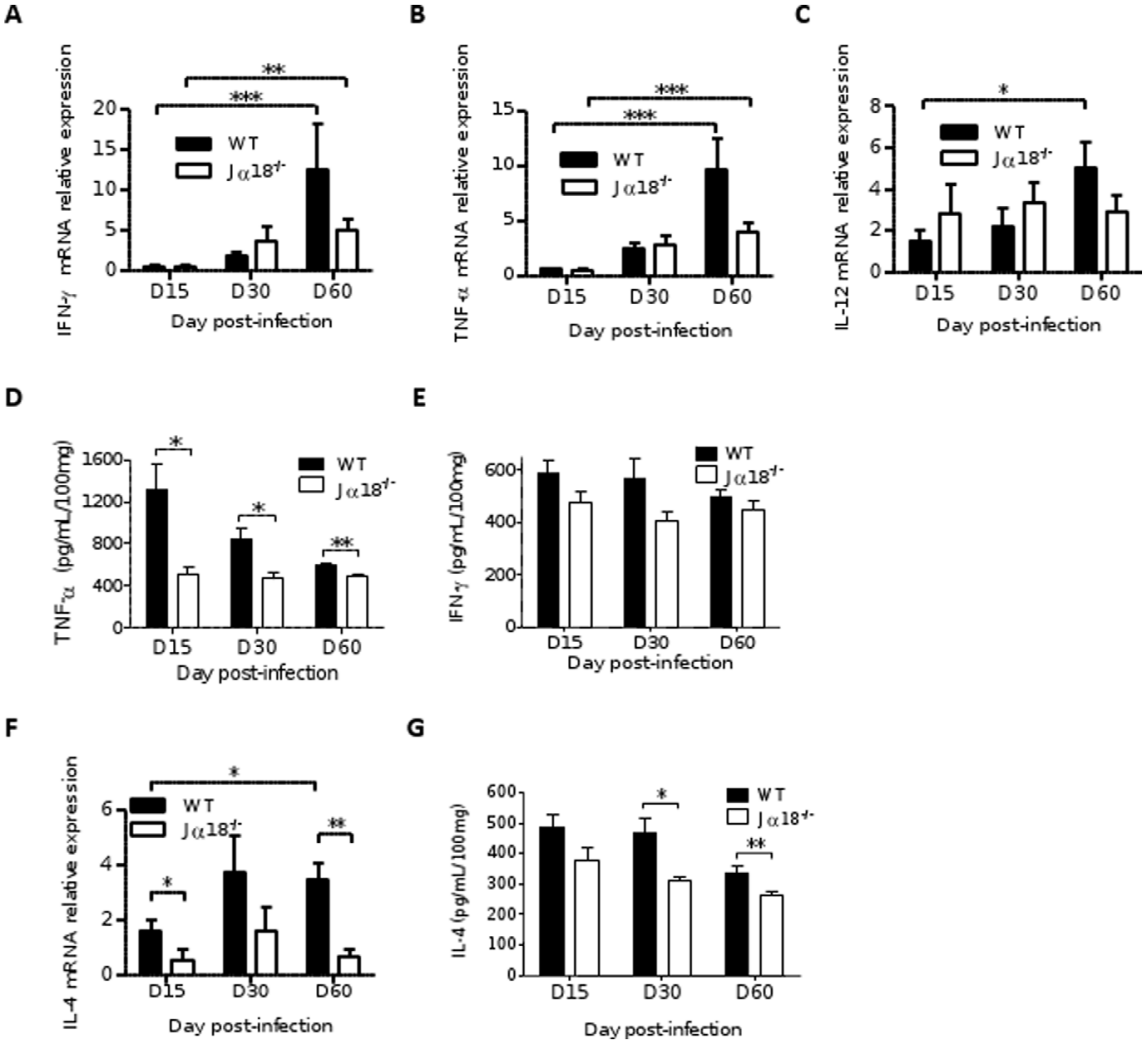
Kinetics of hepatic mRNA induction and expression of key cytokines in WT and Jα18^-/-^ mice infected with *L.donovani*. Quantification of mRNA induction of IFN-γ (A), TNF-α (B), and IL-12 (C). Quantitative PCR was performed in liver extracts at various time points after infection and normalized by comparison to 18S mRNA. (D) Detection of TNF-α by ELISA in liver extracts. (E) Quantification of mRNA induction of IL-4. (F) Detection of IL-4 by ELISA in liver extracts. Data are the mean±SEM for each group of mice (8–10 mice per group from two independent experiments).

As efficient granuloma formation is under the control of key pro-inflammatory cytokines, such as IFN-γ, IL-12, and TNF-α [10], [12], [13], we focused on individual analysis of mRNA expression of these genes. All three cytokines increased significantly during the course of the infection in WT mice, reaching a maximal expression level on D60 (p<0.05, p<0.001, and p<0.001, respectively). In Jα18^-/-^ mice, IFN-γ and TNF-α, but not IL-12, were also induced at significant levels on D60 compared to D15 (p<0.01 and p<0.001, respectively, Figure 6A–C). Although the mean induction rate was higher for all three cytokines in WT mice on D60, this difference did not reach statistical significance when compared to Jα18^-/-^ mice. Overall, the number of granulomas was highly correlated with mRNA induction of IFN-γ (r = 0.82, p<0.0001) and TNF-α (r = 0.83, p<0.0001). In addition, TNF-α was highly correlated with the number of large granulomas (r = 0.85, p<0.0001) (Table 1).

**Table 1:** Spearman’s correlation rank between variables.

	Granulomas	Large granulomas	MPO^+^ cells/100 fields	TNF-α mRNA	MPO mRNA	iNOS mRNA
IFN-γ mRNA	0.82****	-	NS	-	-	-
TNF-α mRNA	0.83****	0.85****	0.71****	-	-	-
CCL2 mRNA	0.84****	0.82****	0.74****	-	-	0.90****
MIP-2 mRNA	0.67****	0.63****	0.71****	-	-	0.85****
iNOS mRNA	0.78****	-	-	-	-	-
MPO^+^ cells	0.85****	0.84****	-	0.71****	0.68****	-
IL-4 mRNA	0.61****	-	-	-	-	-
MPO mRNA	0.65****	-	0.68****	-	-	-
IL-10 mRNA	0.52****	-	-	-	-	-
KC mRNA	0.52****	-	0.38**	-	-	-
KC ELISA	0.47***	-	0.38**	-	-	0.74****
IL-12 mRNA	0.4** a	-	-	-	-	-
Liver weight	0.47***	0.53****	-	-	-	-
LDU	NS b	NS	- 0.32* c		- 0.49*** d	NS e

NS, not significant; *, p<0.05; **, p<0.01; ***, p<0.001; ****, p<0.0001.

a NS in Jα18-/-in individual analysis.

b −0.53 (p<0.05) in WT, NS in Jα18-/-mice.

c −0.52 (p<0.01) in WT, NS in Jα18-/-mic.

d −0.74 (p<0.0001) in WT.

e −0.41 (p<0.05) in WT.

In liver extracts, TNF-α was detected by ELISA at higher concentrations in WT mice at all time points (p<0.05 on D15 and D30, and p<0.01 on D60), but the level of hepatic IFN-γ did not differ significantly between the two groups of mice, although it was higher at all time points in WT (Figure 6D–E).

### IL-4 detection is higher in the liver of wild-type mice and its induction is reduced in Jα18^-/-^mice

A striking difference in the expression and induction of IL-4 was observed between the two groups of mice. IL-4 induction increased over time in WT mice only (p<0.05) (Figure 6F), whereas it was usually absent in Jα18^-/-^ mice, which displayed induction indices at lower levels than in control non-infected mice. IL-4 mRNA induction was highly correlated to IL-12 induction in WT (r = 0.72, p<0.0001). The detection of IL-4 in the liver tissue by ELISA was also significantly higher in WT mice compared to Jα18^-/-^ mice on D30 (p<0.05) and D60 post-infection (p<0.01) (Figure 6G).

### Chemokine mRNA expression is sustained and raises over time in WT mice

As chemokines are powerful chemoattractants for several cell types known to contribute to granuloma assembly, the transcriptomic approach used at early time points (Table S1) was also applied on late time points. It appeared that expression of most chemokines was sustained over time in WT animals and peaked at D60. By contrast, 11/13 chemokines and 6/7 receptors for chemokines were under-expressed in Jα18^-/-^ mice at D60 compared to WT mice, as shown in Figure S2, which displays the most significant differences of gene expression (i.e. differences in mRNA induction of at least 2-fold between the two groups of mice).

**Figure 7:**
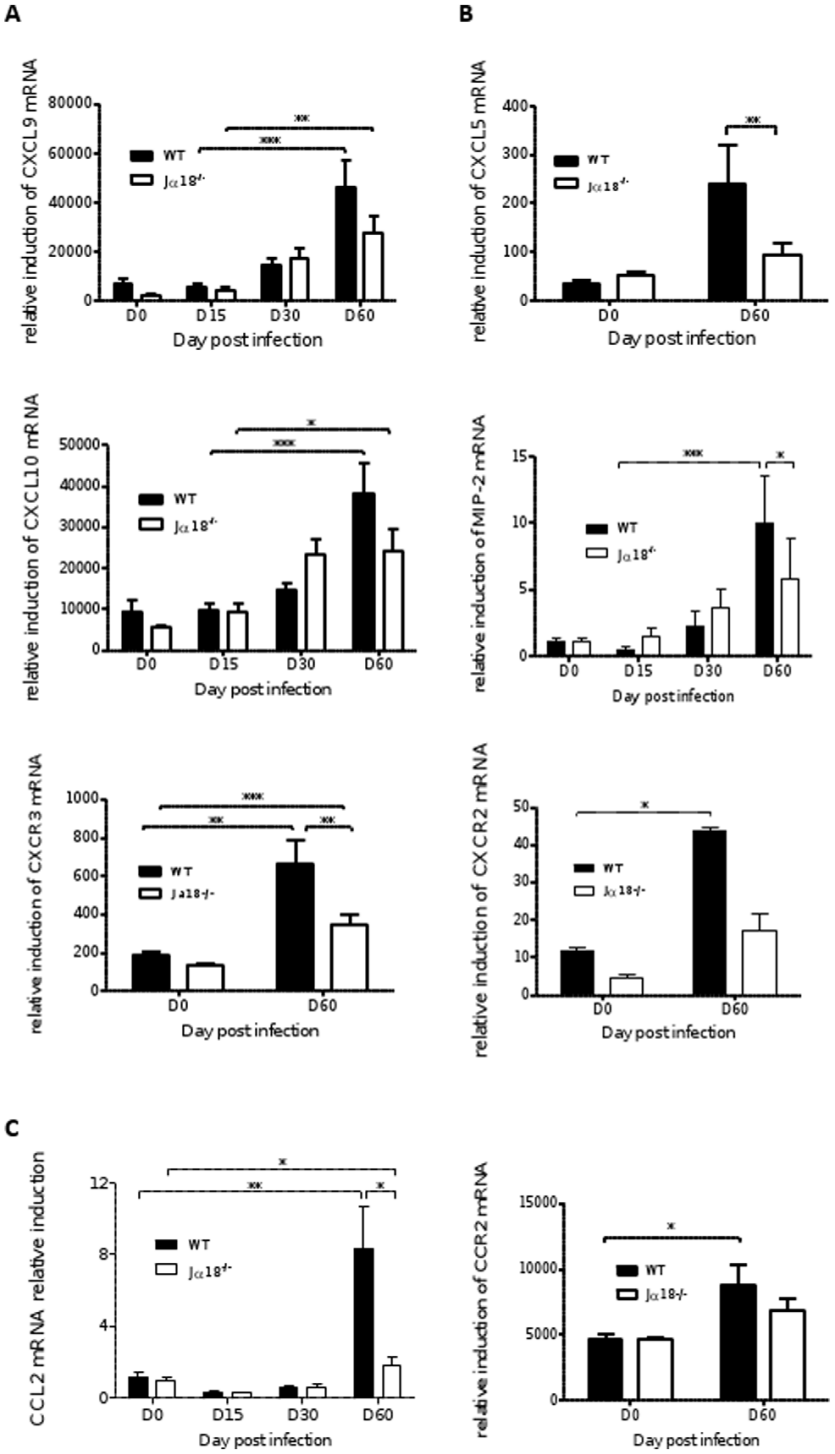
Kinetics of selected chemokine and related chemokine receptors expression in liver extracts from WT and Jα18^-/-^ mice. mRNA expression of selected chemokines and their receptors was quantified by qPCR in liver extracts at various time points after infection. Quantification of mRNA for the T and NK cell-chemoattractant chemokines CXCL9, CXCL10 and their receptor CXCR3 (A), quantification of the PMN-chemoattractant chemokines MIP-2 and CXCL5 and their receptor CXCR2 (B) and of CCL2 and its receptor CCR2 (C). Data are the mean±SEM of 8 to 10 mice per group from two independent experiments and normalized on the expression of three housekeeping genes.

Quantification of gene expression of some of the most expressed chemokines (compared to housekeeping genes) was made after individual analysis of cDNA from all mice. Whereas the T cell and NK-chemoattractant chemokines CXCL9 and CXCL10 were strongly induced over time in both groups of mice, the induction of their receptor CXCR3 was higher in WT mice (p<0.05, Figure 7A), which is consistent with the data obtained by flow cytometry analysis. The neutrophil-chemoattractant chemokines MIP-2/CXCL2 and CXCL5 [37], as well as their receptor CXCR2 were strongly induced on D60 in WT mice only (Figure 7B). Similarly, CCL2/MCP-1 and its receptor CCR2 were also higher on D60 in WT mice than in Jα18^-/-^ (p<0.05) (Figure 7C). There was a strong correlation between mRNA induction of CCL2 and MIP-2 and the number of granulomas (r = 0.84, and r = 0.67, respectively; p<0.0001). Similarly, CXCL1/KC induction was also correlated, to a lesser degree, with the number of granulomas (r = 0.52, p<0.0001) (Table 1).

### Myeloid cell infiltration is strongly reduced in the liver of infected Jα18^-/-^ mice

**Figure 8:**
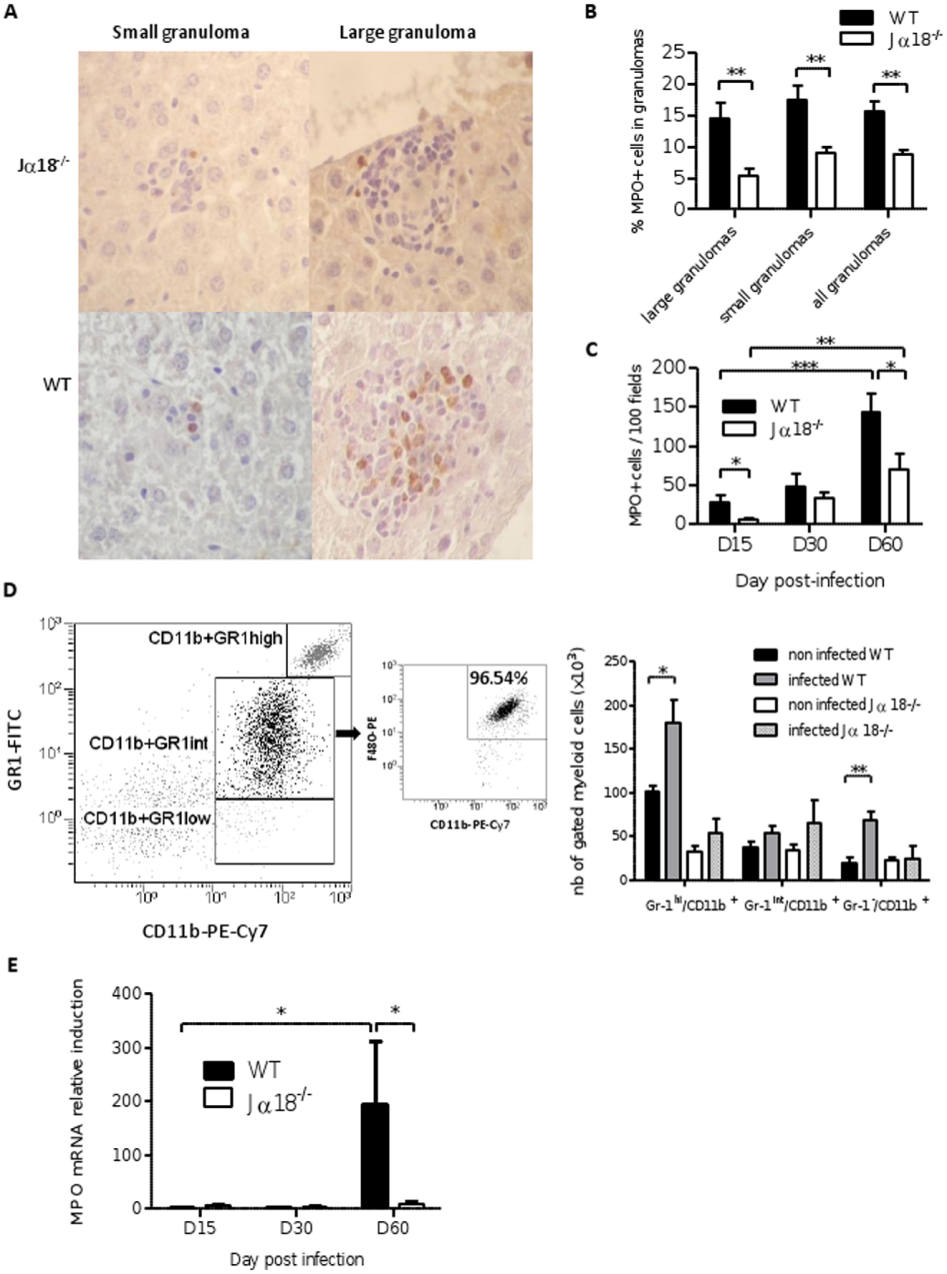
Quantification of myeloid cells in the liver of wild-type and Jα18^-/-^ mice. (A) Representative slides of small (Day 15) and large (Day 60) hepatic granulomas labeled by immunohistochemistry using an anti-MPO antibody, in WT and Jα18^-/-^ mice. (B) Proportion of MPO^+^ cells in granulomas, according to the size of the foci (i.e. small granulomas of <25 cells, and large granulomas >25 cells). (C) Quantification of the absolute number of MPO^+^ cells in granulomas in 100 consecutive microscopic fields (×400) at various time points. Data are the mean ± SEM for each group of mice (8–10 mice per group from two independent experiments). (D) Absolute quantification of myeloid cell infiltration in the liver of C57BL/6 WT and Jα18^-/-^ by flow cytometry on D60 after infection with *L. donovani*, using an anti-Gr1-FITC, anti-CD11b-PE-Cy7, and anti-F4/80-PE. Analysis was done on live cells after excluding lymphocyte cells. 10^6^ cells of liver homogenates were labeled and data were analyzed on 50.000 events. Data are the mean ± SEM of four mice per group. (E) Quantification of MPO mRNA expression in the liver of WT and Jα18^-/-^ mice. Data are the mean±SEM for each group of mice (8–10 mice per group from two independent experiments).

Since striking differences in expression were observed for chemokines attractant for neutrophils (CXCL1, MIP-2/CXCL2, CXCL5) or monocytes (CCL2/MCP-1) on late time points [37], [38], we quantified these cells within granuloma by immunohistochemistry using an anti-myeloperoxidase (MPO) antibody which mainly stains PMN cells (Figure 8A). Not surprisingly, we obtained contrasting data between WT and Jα18^-/-^ mice, with higher proportion of MPO-positive cells within the granulomas of WT mice, whatever the size of granulomas (p<0.01) (Figure 8B).

Consistent with chemokines expression data, the absolute number of intra-granuloma MPO-positive cells counted in 100 consecutive microscopic fields (×400) increased during the 60-day follow-up (p<0.001 and p<0.01, for WT and Jα18^-/-^ mice, respectively) (Figure 8C). Moreover, this cell attraction was correlated with the total number of granulomas (r = 0.85; p<0.0001) (Table 1). MPO-positive cell number was about 2-fold higher in WT mice compared to Jα18^-/-^ mice on D60 (144±23 and 70±20, respectively; p<0.05) and 5-fold higher on D15 (28±9 vs 5±1, respectively; p<0.05) (Figure 8C).

The level of MPO-positive cell infiltration was highly correlated with mRNA induction of MIP-2 and CCL2 chemokines (Spearman r = 0.71 and r = 0.74, respectively; p<0.0001), and to a lesser extent to mRNA induction of CXCL1/KC (Spearman r = 0.38; p<0.01) (Table 1). When performing the statistical analysis on each individual group of mice, induction of CXCL1/KC was correlated with the number of MPO-positive cells in WT mice (r = 0.58; p<0.01) but not in Jα18^-/-^ mice.

Immunohistological findings were confirmed by flow cytometry analysis of hepatic immune cells on D60, which clearly demonstrated that PMN cells (GR-1^hi^/CD11b^+^ panel) were present in larger amounts in WT than in Jα18^-/-^ mice (180±25 ×10^3^ versus 54±16 × 10^3^ cells; p<0.05) representing more than half of the myeloid cell population at that time (Figure 87D–E). Macrophage cells (GR-1^int^/CD11b^+^ subset, which matched with F4/80+ cells) accounted for about 18% of myeloid cells and did not rise significantly (Figure 8D–E). An increase in monocyte cell counts (GR-1^low^/CD11b^+^ subset) was also observed on D60 (p<0.01) in WT mice, reaching about 23% of the amount of myeloid cells (Figure 8D). By contrast, no significant change in the amount of myeloid cell subsets was noted in Jα18^-/-^ mice, which is consistent with the defect in chemokines observed in these mice.

**Figure 9:**
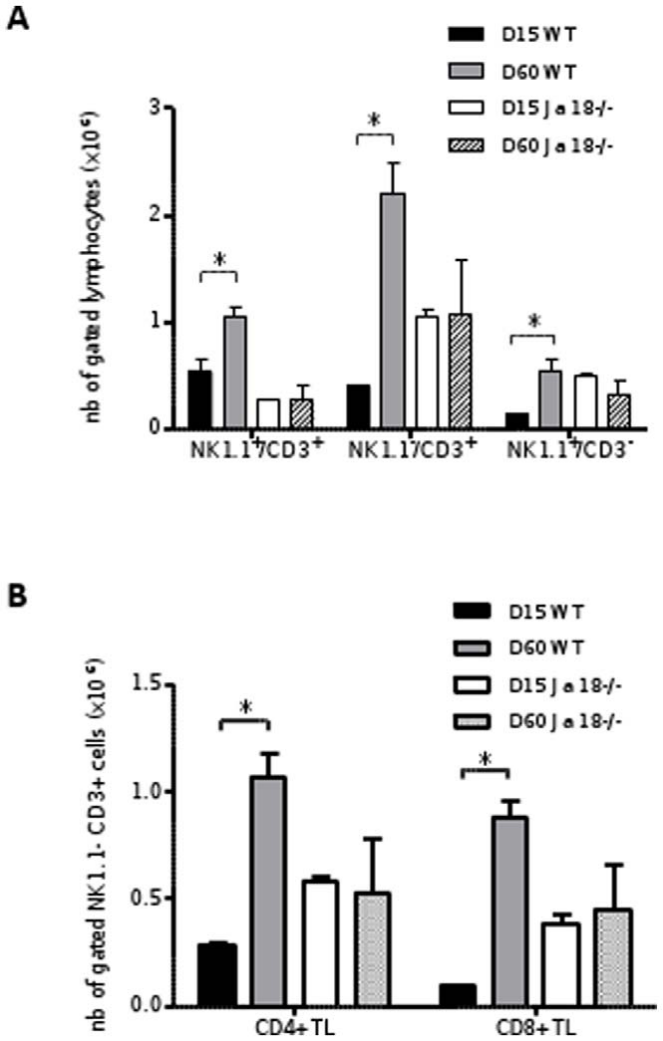
Hepatic lymphoid cell infiltration in wild-type and Jα18^-/-^ mice at day 60 of infection. Quantification of lymphoid cells in the liver of C57BL/6 WT and Jα18 by flow cytometry after infection with *L. donovani*, using anti-CD4-PE, anti-CD3-Pacific Blue, anti-NK1.1-PerCP-Cy-5.5, and anti-CD8-APC-Cy7. 10^6^ cells of liver homogenates were labeled and data were analyzed on 50.000 events. Absolute quantification of hepatic NK (NK1.1+/CD3-), NKT (NK1.1+/CD3+), and TL (NK1.1-/CD3+) cell subsets (A), absolute quantification of CD4^+^ and CD8^+^ cell subsets in gated TL (B). Data are the mean ± SEM of four mice per group.

Concomitant analysis of lymphoid cell subsets revealed also a sustained attraction of all three subsets, i.e. NKT cells, T lymphocytes and NK cells (p<0.05) from D15 to D60 in WT, with a large predominance of T lymphocytes, whether CD4^+^ or CD8^+^ (Figure 9A–B). These results are indeed in accordance with a progressive maturation of granulomas.

### Phagocytic enzymatic effectors are reduced in Jα18^-/-^ mice

The final step of granuloma maturation in the liver is the potentiation of phagocytic cells to eliminate the parasite through ROI and RNI intermediates. The induction of inducible nitric oxide synthase (iNOS) and of MPO genes was measured in liver extracts. MPO induction increased throughout the infection in WT mice (p<0.05 between D15 and D60) but not in Jα18^-/-^ mice (Figure 8F). The induction of MPO mRNA was correlated with MPO-positive cell infiltration and with the number of granulomas (Spearman r = 0.68 and r = 0.65, respectively, p<0.0001), and inversely correlated with hepatic parasite burden (Spearman r = −0.49, p<0.001), this correlation being stronger in WT (Table 1). The induction of iNOS was also correlated with the granuloma tissue response (Spearman r = 0.78, p<0.0001), and was higher on D60 compared to D15 post-infection (p<0.01). Like other markers, iNOS induction was highly correlated with MIP-2 and CCL2 induction (r = 0.85 and 0.90, respectively; p<0.0001), as well as with CXCL1/KC (r = 0.74; p<0.0001). Moreover, iNOS induction was correlated to parasite clearance in WT mice only (Table 1).

## Discussion

NKT cells together with dendritic cells are pivotal cell subsets involved in the link between innate and adaptive immune response against microorganisms. A role for NKT cells in the defense against *Leishmania donovani* infection was pointed previously by Amprey *et al*. [26], who showed that liver granuloma formation was impaired and parasite burdens were higher in the absence of NKT cells, using a model of BALB/c CD1d^-/-^ mice. However the genetic background is known to influence the capacity of mice to control *Leishmania* infection [39], and BALB/c mice which display a biased Th2 immune response, express a different susceptibility profile to infection. Moreover, among NKT cells, the precise role of the iNKT cell subset, the main T cell subset in the mouse liver, needed to be confirmed, since contradictory data were reported by Stanley *et al*. [33]. In the aim to display robust conclusions about the requirement of these cells for parasite control in the liver, we used a model of iNKT cell-deficient mice on a C57BL/6 genetic background, a more resistant genetic background due to its capacity to produce more IFN-γ than BALB/c mice [40]. Hence, enhanced susceptibility in the absence of iNKT cells would strongly suggest that these cells actively participate to mouse resistance. We showed effectively that WT mice drove efficient parasite clearance after infection. Conversely, parasite proliferation was significantly higher in the liver of Jα18^-/-^ mice at all time points post-infection. Our model of intraperitoneal inoculation of promastigotes yielded lower parasite burdens in the liver than those reported in other studies using amastigotes by intravenous route, but we speculate that the intraperitoneal route is closer from the natural inoculation of promastigotes by sand flies. In their study, Stanley *et al*. [33] also found higher parasite loads in the liver of Jα18^-/-^ mice on late time points (D56) after infection with *L. donovani*. However, the authors concluded to a minor physiological role in the protection during murine *Leishmania* infection, since cell stimulation by injection of α-GalCer prior or concomitantly to mouse infection did not concur to improve disease resolution, but even led to enhanced parasite burdens [33]. In our opinion, this observation, under non physiological conditions, should warn on the possible risk of a paradoxical effect during NKT cell activation, but cannot allow to draw definitive conclusions about the role of iNKT cells during the natural course of infection. In other models, activation of iNKT cells with α-GalCer led to contradictory results, suggesting that over-stimulation does not reflect the fine regulation of these cells *in vivo* in the context of physiological activation, since it does not reproduce exactly the microenvironment associated with antigen presentation. For example, in the work of Osman *et al*. [41] activation of hepatic NKT cells with α-GalCer induces liver injury, whereas this cell subset is protective against acute hepatitis in another study [42]. Another important point is that strong activation of iNKT cells with α-GalCer can lead to a profound anergy of these cells and a down-regulation of CD1d [43], which renders them refractory to subsequent activation, and could explain the exacerbation of *Leishmania* infection observed by Stanley *et al*. [33].

The better control of parasite burdens observed as soon as day 15 in WT mice was associated with a massive iNKT cell infiltration detected by flow cytometry in the liver. Indeed, the TCRβ+/CD1d-αGalCer-TT+ cell subset accounted for the dramatic increase of CD3+/NK1.1+ cells in WT mice, whereas other minor subsets remained almost stable (Figure 4). In addition, iNKT cells supported most of the CXCR3 and CD69 expression in lymphoid cells (Figure S3 and data not shown), suggesting that they are the activated T cell main subset recruited at this time point. A recent Indian study conducted in Human patients infected with *L. donovani* pointed to an increased amount of iNKT cells both in peripheral blood and bone marrow compared to healthy controls [44], as well as an increased proportion of the whole CD69+ NKT cell subset in the bone marrow from patients with visceral leishmaniasis [45], suggesting a role in the defense against the parasite. The proportion of iNKT cells returned to basal levels after treatment [44].The CD4^+^ iNKT cell subset was the predominant infiltrating subset in WT mice; this cell subset was previously reported to be crucial in the control of parasite burdens in lymph nodes from C57BL/6 mice during the first week following infection with *L. major* [34], [46]. We also showed the presence of a NK1.1-/CD1d-αGalCer-TT+ iNKT cell subset, which role remains to be determined. However, its proportion was unchanged after infection.

The higher levels of hepatic parasite burdens in Jα18^-/-^ mice were associated with a defect in the process of tissue granulomas formation. The results observed during our two-month follow-up of WT and Jα18^-/-^ infected mice suggest that iNKT cells contribute to a favorable cytokinic/chemokinic environment that allows an efficient lymphoid response at early steps, but also influence late steps of the granuloma maturation process. We postulate that iNKT cells are the conductor of a progressive orchestration of synchronized actors through progressive and sustained chemokines synthesis, modulating the recruitment and function of various cell types. It is long known that pro-inflammatory cytokines play a key role during the course of infection. TNF-α, was detected by ELISA at significantly higher concentrations in the liver of WT mice at all time points. These data are consistent with a sustained hepatic impregnation with TNF-α, reflecting the greater amount and stronger responsiveness of inflammatory cells in WT animal. TNF-α has previously been shown to play a pivotal role in T cell and monocyte recruitment [5], and Murray *et al.* [12] observed in TNF-α knock-out (KO) mice, that the absence of granuloma assembly on D15 post-infection was correlated with a higher liver parasite burden. Both IL-12 and IFN-γ are also required for resistance to *Leishmania* infection and granuloma assembly, as demonstrated in studies using KO mice [47], [48] or anti-IFN-γ or anti-IL-12 injections [8], [10]. In the present study, IL-12 induction increased significantly between D15 and D60 post-infection, and was correlated with the number of granulomas in WT mice only. Murray [3] reported that there was a defect in granuloma formation in IL-12 and IFN-γ KO mice, with a “no structure-no function” relationship, extended to the later stage of infection. Thus, the partial deficiency of IL-12, IFN-γ, and TNF-α induction in Jα18^-/-^ mice could be responsible for a similar mechanism, since the granulomas observed in these mice were less abundant and less well-organized than in WT mice. Interestingly, IFN-γ expression increased throughout the infection and was strongly correlated to the number of granulomas, irrespective of the type of mice, despite higher levels of induction in WT mice. This observation raises the possibility of partial compensatory mechanisms for IFN-γ production in Jα18-/-mice, probably through T cells or NK cells, but inadequate to drive efficient chemokines pathways. A recent study suggests that maximal activation of NK cells would result from a cross-talk between NK and iNKT cells, thus would be observed only in WT mice. Indeed, it was shown that iNKT activation by α-GalCer results in a high speed transactivation of NK cells in a model of C57BL/6 mice, as well as a delayed activation of B cells and T cells [49].

Recently, Uemura *et al*. [50] demonstrated that stimulation of iNKT cells by their specific ligand α-GalCer modifies the IL-12p70/IL-23 balance in favor of enhanced IL-12p70 production by dendritic cells; the down-regulation of IL-23 was markedly associated with IL-4 and IL-10 production by iNKT cells. These data corroborate our results, since we detected significantly higher levels of IL-4 by ELISA and mRNA in WT than in Jα18^-/-^ mice, together with a significant induction of IL-12 in WT mice only, both cytokines being highly correlated in these mice (r = 0.71, p<0.0001). These observations are also consistent with the data of Zhu *et al*. [29], who showed that in a model of T cell mediated hepatitis, the capacity of iNKT cells to produce IL-4 was directly influenced by IL-12. Altogether, these data could point iNKT cells as a major hepatic source of IL-4, contributing to the peculiar hepatic microenvironment and involved in the development of efficient granuloma maturation [13].

Granuloma maturation is indeed a complex process depending on efficient attraction of competent cells in the aim to help infected cells to get rid of parasites. To explain the strong and sustained attraction of immune cells in the liver of infected mice all along the infection process, we investigated large scale chemokine expression, and found that WT mice were more likely able to produce most chemokines, particularly Th1 chemokines, and to induce their receptors than Jα18^-/-^ mice. In particular, we showed that IFN-γ-dependent chemokines (CXCL9/10) were strongly induced, as shown by others [51], and increased during the course of infection, as well as their receptor CXCR3. The role of NKT cells in the homing of CXCR3+ cells in the liver was described in a recent paper [52], but in our model iNKT cells themselves represent the main proportion of CXCR3+ cells infiltrating the liver. Here we also show that numerous other chemokines were generally more strongly induced in WT compared to Jα18^-/-^ mice. Whereas lymphoid cells-attracting chemokines (CCL19, CCL27, CXCL16, CCL9, CCL25) were significantly induced at early time points, myeloid cells-attracting chemokines are mostly induced on D60. Among them, we observed in WT animals a marked expression of the PMN-attractant chemokines MIP-2/CXCL2 and CXCL5, and of the monocyte-attractant chemokine CCL2/MCP-1, as well as of their receptors CXCR2 and CCR2, respectively. This strong induction was correlated with the number of granulomas and in particular with mature granulomas, therefore is supposed to help resolving infection. In cutaneous leishmaniasis, a number of chemokines were shown to be expressed in Humans, of which CCL2, CCL19 and CCL17 [53]. An essential role for CCL2 in the defense against intracellular pathogens, was also described for *Listeria* [54]. Consistent with expression data, flow cytometry analysis of liver immune cell populations assessed the presence of significantly higher amounts of granulocytes and monocytes in WT animals on D60. Histological data definitely confirmed the progressive and stronger attraction of MPO-labeled cells within the hepatic granulomas of WT mice. Finally, we demonstrated the higher induction of enzymes of the oxidative burst, iNOS and MPO, in WT mice, and their correlation to the efficient control of parasite burdens. MPO activity is mostly due to neutrophils, whereas iNOS is associated to phagocytic potency of monocytes-macrophage cells which are target cells for *Leishmania* parasites and display enhanced phagocytic activity after activation. Recent data have shown that neutrophils contribute to efficient protective immune response during *L. donovani* infection of BALB/c mice, since in neutrophil-depleted mice, parasite burdens were higher and granuloma formation was impaired [55]. In Human, the lack of neutrophils recruitment is a histopathologic feature of diffuse cutaneous leishmaniasis, by contrast to local cutaneous leishmaniasis [53], which is also an indirect argument pleading for a beneficial role of these cells. Another recent paper [56] is in accordance with our observations, since it claims that activation of iNKT cells ameliorates listeriosis by accelerating infiltration of Gr-1^+^ cells into the liver. However, the role of neutrophils in the late stage of hepatic infection with *L. donovani* and the accurate link between iNKT cells and neutrophil attraction in the liver are poorly explored, and should be revisited in the light of these results.

Many chemokines were shown to be induced in the liver of WT mice in our study. They probably have intricate and redundant actions and because of the long term follow-up of this study design, it was not possible to dissect their role independently, as it can be made in early steps of infection. However, this large scale descriptive study offers an overview of chemokine expression and provides new insights on the complex pathophysiology of granuloma assembly in the liver. Finally, our data are consistent with a role of iNKT cells in the efficient host response and early control of *L. donovani* proliferation.

## Materials and Methods

### Ethics statement

This study was carried out in accordance with the French institutional guidelines (Direction des Services Vétérinaires, agreement number 35–86), and with the EC directive #86/609/CEE. The use of Jα18^-/-^ mice in our animal facilities was approved by the Commission Génie Génétique (Ministère de l’Enseignement Supérieur et de la Recherche, agreement number 5387-CA-1).

### Mice

Female C57BL/6 wild-type mice were purchased from Janvier Laboratories (Le Genest-Saint-Isle, France) and acclimatized for at least 10 days before challenge. C57BL/6 iNKT cell-deficient (Jα18^-/-^) mice were generated and provided by M. Taniguchi (Chiba University, Japan) and were backcrossed for at least 10 generations. Mice were bred and housed in our animal facilities under pathogen-free conditions. Mice were 7–10-weeks-old when challenged with *L. donovani*. Naïve congenic mice, matched according to age, were used as non-infected controls.

### Parasites and infection of mice

The *L. donovani* strain (MHOM/SD/97/LEM3427, typed as Zym MON-18 by the Centre National de Référence des Leishmanioses, Montpellier, France) was maintained *in vivo* by serial murine passages and grown *in vitro* on NNN (Novy-McNeal-Nicolle) blood agar at 27°C. Prior to infection, amplification of promastigotes was carried out by culture in Schneider’s *Drosophila* medium (Invitrogen, Carlsbad, CA) supplemented with 10% FCS, 100 U/mL penicillin and 100 µg/mL streptomycin, for 6 days at 27°C, until they reached stationary phase. Animals were infected on day 0 (D0) by intraperitoneal injection of 10^8^ promastigotes, and groups of 4–6 mice were sacrificed on D15, D30, or D60. Prior to sacrifice, blood was collected by retro-orbital puncture, and the serum was stored at –80°C. The liver was recovered and weighed, then cut into pieces, formalin-fixed and paraffin-embedded or snap frozen in isopentane/liquid nitrogen for mRNA and protein extraction.

### Quantification of liver parasitic burden

Parasite burden was determined by microscopic examination of Giemsa-stained smears, with the results expressed as Leishman Donovan Units (LDU) (i.e. number of amastigotes per 1000 cell nuclei×liver weight in mg).

### Quantification of granulomas in liver tissue

The histological response to infection was evaluated quantitatively after microscopic examination of paraffin-embedded liver sections stained with H&E. Granulomas were classified into two categories based on the number of cells: i) <25 cells; ii) >25 cells. The type of granuloma assembly was also recorded, i.e. mature granuloma foci displaying a well-organized peripheral ring of lymphocytes surrounding a Kupffer cell core, or poorly organized granuloma foci.

### Immunohistochemical characterization of liver granulomas

The attraction of some effector cells to granulomas was quantified after immunohistochemical staining. T lymphocytes were stained using an anti-CD3 rabbit monoclonal antibody diluted 1/200 (Thermo Fisher Scientific, Fremont, CA, USA) and PMN were stained using a polyclonal rabbit anti-myeloperoxidase (MPO) antibody diluted 1/2000 and a Vectastain ABC kit (Vector Laboratories, Peterborough, UK), according to the manufacturer’s instructions. The sections were counterstained with hematoxylin.

### RNA isolation and analysis of hepatic gene expression

Total cellular RNA was extracted and purified from liver samples using Trizol reagent (Invitrogen), then treated with DNase (10 U DNase I/µg total RNA) and reverse transcribed with SuperScript^TM^ II Reverse Transcriptase (Invitrogen) according to the manufacturer’s instructions. A screening of a large panel of cytokines and chemokines and their receptors was analyzed (listed in Table S1) using a large-scale qPCR approach to analyze the global response of liver cells to infection. PCR amplification reactions were carried out in triplicate with iQ-SYBR Green Supermix (Bio-Rad) in a 15µl reaction volume containing 200 nM primers and 5 ng of pooled cDNA from each time point and group of mice, using a Chromo4™ System (Bio-Rad). Primers were designed using Primer3 software (http://frodo.wi.mit.edu/cgi-bin/primer3/primer38www.cgi) and the corresponding sequences will be available upon request. The ΔCt method was retained for quantification and GAPDH, HPRT1 and HSPCB housekeeping genes used for multiple normalization as described previously [57].

Quantitative analysis of expression of selected genes was performed using the StepOne plus system (Applied Biosystems, Cergy-Pontoise, France) in 96-well optical plates with Power SYBR®green PCR Master Mix (Applied Biosystems), 3 µM primers and cDNA corresponding to 30 ng of total RNA input in a final volume of 20 µL. Samples were analyzed in duplicate and expression levels of target genes were normalized to expression of 18S rRNA. Results were expressed as 2^–ΔΔCt^ referred as fold induction in relation to the mean Ct obtained with non-infected WT mice. The PCR primers were designed using Primer express 3 software and synthesized by Qiagen or Sigma-Aldrich (Lyon, France).

### Tissue cytokine assay

For the measurement of hepatic IL-4, TNF-α, and IFN-γ expression, liver samples (200 mg) were homogenized individually using a disperser (Ultra-Thurax T25; Janke & Kunkel, Labortechnick, Germany) in 0.5 ml of ice-cold PBS containing complete protease inhibitor cocktail (Roche Applied Science, Meylan, France). Homogenates were centrifuged for 15 min at 10,000 rpm at 4°C. The supernatants were recovered and submitted to a second centrifugation. All lysates were equalized with complement-free FCS to a 10% concentration, and tested whole and diluted 1∶2 in 10% complement-free FCS. The different cytokines were quantified using Duoset ELISA development systems for mouse IL-4, TNF-α, and IFN-γ (R&D Systems), according to the manufacturer’s instructions, except for the reveal step in which orthophenyl-dianizidine was used instead of tetramethylbenzidine. Absorbance was determined at 490 nm using a spectrophotometer, and the results were determined from a seven-point standard curve, and expressed as pg/mL/100 mg of tissue.

### Flow cytometric analyses

For flow cytometric analyses, livers were perfused with PBS to remove blood cell contamination prior to dissection. After homogenization of liver tissue and elimination of hepatocytes by sedimentation, immune cells were purified using 35% Percoll (GE healthcare) and red blood cells were lyzed. 10^6^ leucocytes were incubated with anti-CD16/32 (BD Pharmingen) to block non-specific binding and washed. The cells were then incubated with appropriate dilutions of anti-Gr1-FITC, anti-CD11b-PE-Cy7, anti-CD11c-APC, anti-CD4-PECy7 or anti-CD4-PB, anti-CD3-PB or FITC, anti-NK1.1-PerCP-Cy-5.5, anti-F4/80-PE, and anti-CD8-APC-Cy7 antibodies, all purchased from BD PharMingen. Other antibody panels associating CD3-V500, anti-TCRβ-V450, anti-TCRγδ-FITC and CD69-PE or CXCR3-PE were used for accurate phenotyping of lymphoid cells. Invariant NKT cells were characterized using αGalCer-tetramer-PE or APC, provided by the NIH tetramer facility (Atlanta, GA). The cells were washed, fixed in PBS containing 2% FCS, 0.01 M sodium azide and 2% formaldehyde. The stained cells were analyzed on a FACS Aria II ® flow cytometer using BD FACS Diva software (BD Bioscience) and the data were processed using CXP software (Beckman Coulter). Dead cells and doublet cells were excluded on the basis of forward and side scatter.

### Statistical analysis

Data are expressed as mean±SEM for each group of mice (8–10 mice per group from two independent experiments). Differences between groups were analyzed using non-parametric tests (Mann-Whitney test). Correlations between variables were evaluated using the Spearman rank correlation test. Paired statistical analysis of mRNA induction between the two groups was performed using the Student’s *t*-test. Data from mRNA high throughput screening were analyzed using the Kruskal-Wallis test and the Dunn’s multiple comparison test. Statistical analysis was performed using GraphPad Prism 5.02 software. Differences were considered significant when the p-value was <0.05, and graduated as *(p<0.05), **(p<0.01), and ***(p<0.001).

## Supporting Information

Figure S1 **Induction rates of genes mRNA from selected immune markers in C57BL/6 and Jα18^-/-^ mice.** (A) Mean induction rates in non infected mice. (B) Mean induction rates 15 days after infection with *L. donovani*. mRNA induction was normalized on housekeeping genes. Gene targets which were not more expressed than housekeeping genes are not represented.
(TIF)

Figure S2 **Ratios of most significant mRNA induction rates of immune markers in Jα18^-/-^ compared to WT mice at early stage (D15) and late stage (D60) of infection (induction of at least +/- 2-fold compared to WT mice).**
(TIF)

Figure S3 **CXCR3 expression on lymphoid cells in the liver of C57BL/6 WT.** Analysis by flow cytometry after infection with *L. donovani*, using, anti-NK1.1-PerCP-Cy-5.5, anti-βTCR-V450, αGalCer/CD1d tetramer-PE, anti-CD4-PE-Cy7, anti-CD8-APC-Cy7, CD3-FITC and anti-CXCR3-APC. (A) Quantification of CXCR3 expression in the following subsets: total lymphocytes, CD4+TL, iNKT cells (TCRβ+/αGalCer/CD1d tetramer+ gated cells), and NK cells (TCRβ-/NK1.1+gated cells). Black transparent curve represents a non infected mice; grey curve represents an infected mice. This panel is representative of three mice per group. 10^6^ cells of liver homogenates were labeled and data were analyzed on 60.000 events. (B) Mean percentage±SEM of CXCR3+cells in each gated cell subset from infected or non infected WT mice. (C) Absolute numbers of recruited CXCR3+cells in the liver from infected or non infected WT mice.
(TIF)

Table S1 **List of immune markers analyzed by transcriptomic approach.**
(DOC)
