# Supplementary figures and images for: Invariant NKT Cells Drive Hepatic Cytokinic Microenvironment Favoring Efficient Granuloma Formation and Early Control of Leishmania donovani Infection
Source: PLoS One. 2012 Mar 22;7(3):e33413. doi: 10.1371/journal.pone.0033413 (PMC3310876; doi:10.1371/journal.pone.0033413)

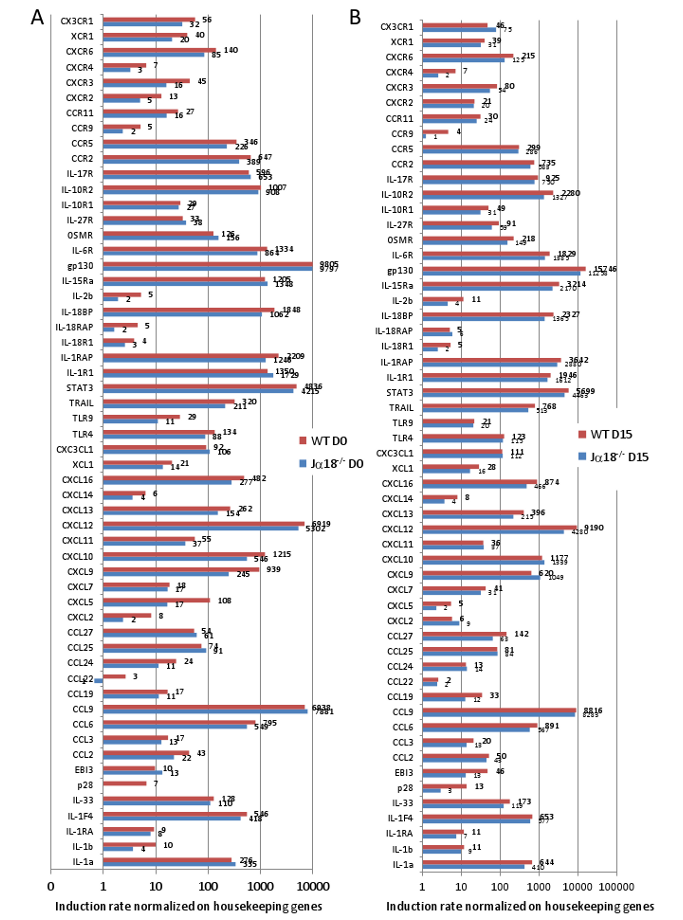

Supplement: Figure S1 — Induction rates of genes mRNA from selected immune markers in C57BL/6 and Jα18-/- mice. (A) Mean induction rates in non infected mice. (B) Mean induction rates 15 days after infection with L. donovani. mRNA induction was normalized on housekeeping genes. Gene targets which were not more expressed than housekeeping genes are not represented. (TIF) [file pone.0033413.s001.tif]

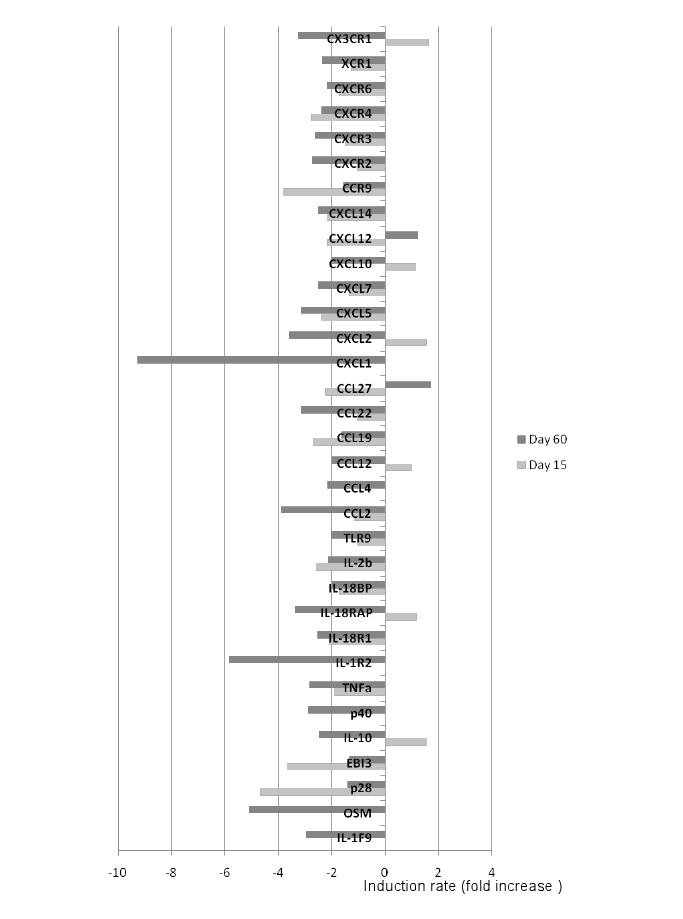

Supplement: Figure S2 — Ratios of most significant mRNA induction rates of immune markers in Jα18-/- compared to WT mice at early stage (D15) and late stage (D60) of infection (induction of at least +/- 2-fold compared to WT mice). (TIF) [file pone.0033413.s002.tif]

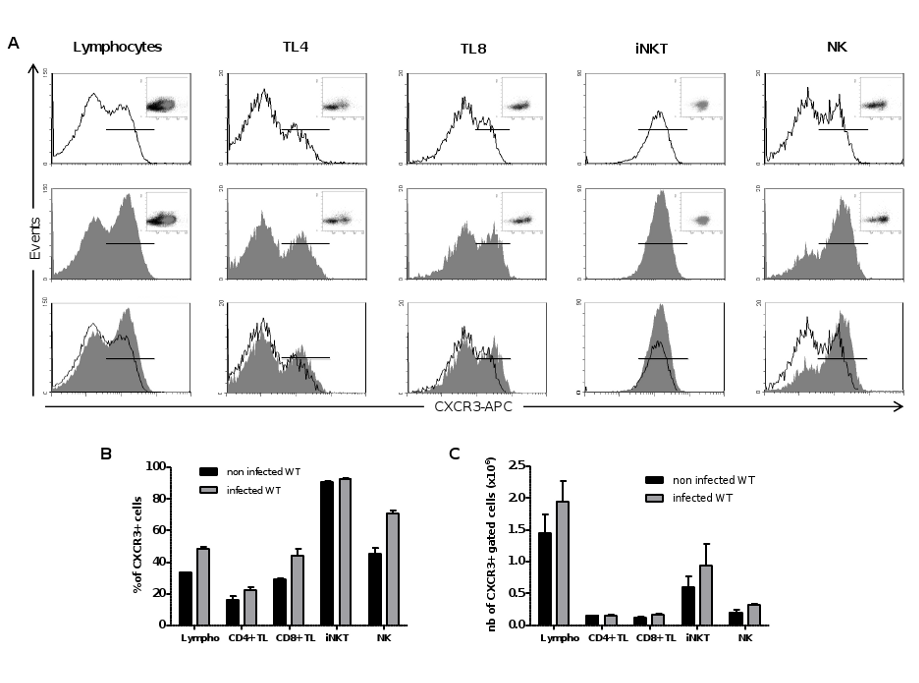

Supplement: Figure S3 — CXCR3 expression on lymphoid cells in the liver of C57BL/6 WT. Analysis by flow cytometry after infection with L. donovani, using, anti-NK1.1-PerCP-Cy-5.5, anti-βTCR-V450, αGalCer/CD1d tetramer-PE, anti-CD4-PE-Cy7, anti-CD8-APC-Cy7, CD3-FITC and anti-CXCR3-APC. (A) Quantification of CXCR3 expression in the following subsets: total lymphocytes, CD4+TL, iNKT cells (TCRβ+/αGalCer/CD1d tetramer+ gated cells), and NK cells (TCRβ-/NK1.1+gated cells). Black transparent curve represents a non infected mice; grey curve represents an infected mice. This panel is representative of three mice per group. 106 cells of liver homogenates were labeled and data were analyzed on 60.000 events. (B) Mean percentage±SEM of CXCR3+cells in each gated cell subset from infected or non infected WT mice. (C) Absolute numbers of recruited CXCR3+cells in the liver from infected or non infected WT mice. (TIF) [file pone.0033413.s003.tif]
