# Supplementary material for: Invariant NKT Cells Drive Hepatic Cytokinic Microenvironment Favoring Efficient Granuloma Formation and Early Control of Leishmania donovani Infection
Source: PLoS One. 2012 Mar 22;7(3):e33413. doi: 10.1371/journal.pone.0033413 (PMC3310876; doi:10.1371/journal.pone.0033413)
Supplement: Table S1 — List of immune markers analyzed by transcriptomic approach. (DOC) [file pone.0033413.s004.doc]

**Supporting Information Table 1 :** List of immune markers analyzed by transcriptomic approach

| **Gene target** | **Functional name / Other name** |
| --- | --- |
| Cytokines |  |
| IL-1α | IL-1F1 |
| IL-1β | IL-1F2 |
| IL-1RA | IL-1F3 |
| IL-1F4 | IL-18 |
| IL-1F5 |  |
| IL-1F9 |  |
| IL-33 |  |
| IL-15 |  |
| TSLP |  |
| IL-6 |  |
| OSM |  |
| p28 |  |
| EBI3 | IL-27B, Epstein-Barr virus- induced gene 3 protein, |
| IL31 | - |
| IL-10 | CSIF |
| IL-22 | IL-10-related T-cell-derived inducible factor (IL-TIF) |
| p40 | IL-12B, CLMF p40, NKSF2 |
| IL-12p35 | IL-12A, CLMF p35, NKSF1 |
| IL-23p19 | - |
| IL-17 | IL-17A, CTLA-8 |
| IL-25 | IL-17E |
| IL-17F | ML-1 |
| IFN-β |  |
| IFN-γ |  |
| TNFα | - |
| Chemokines |  |
| CCL1 | I-309, SCYA1, P500, SISe, (TCA3) |
| CCL2 | MCP-1, SCYA2, MCAF, SMC-CF, GDCF-2, HC11, (JE) |
| CCL3 | MIP-1α, SCYA3, LD78a, AT464.1, G0S19-1, SCI,TY-5, L2G25B, SISa |
| CCL4 | MIP-1β, SCYA4, AT744.1, Act-2, G-26, HC21, H400, SISg, MAD-5 |
| CCL5 | RANTES, SCYA5, SISd |
| CCL6 | C-10, MRP-2 |
| CCL7 | MCP-3, SCYA7, NC28, FIC, MARC |
| CCL8 | MCP-2, MARC,SCYA8, HC14 |
| CCL9/10 | MIP-1γ, MRP-2 |
| CCL11 | eotaxin, SCYA11 |
| CCL12 | MCP-5 |
| CCL17 | TARC, SCYA17, TARC, ABCD-2 |
| CCL19 | MIP-3β, SCYA19, ELC, exodus-3, CKβ11, Scya19-ps1-3 |
| CCL20 | MIP-3α, SCYA20, LARC, exodus-1, ST38, CKβ4 |
| CCL21 | SCYA21, SLC, 6Ckine-leu (Scya21β), exodus-2, TCA4, 6Ckine-ser (Scya21α), CKβ9, 6CKBAC1, 6CKBAC2 |
| CCL22 | SCYA22, MDC, STCP-1, ABCD-1, DC/B-CK |
| CCL24 | eotaxin-2, SCYA24, MPIF-2, CKβ6 |
| CCL25 | TECK, SCYA25, TECK, Ckβ15 |
| CCL26 | eotaxin-3, SCYA26, SCYA26, IMAC, MIP-4α, TSC-1 |
| CCL27 | CTACK, SCYA27, ALP, skinkine, ILC, ESkine, PESKY |
| CCL28 | MEC, SCYA28, CCK1 |
| CXCL1 | GROα, SCYB1, GRO1, MGSA-α, NAP-3 |
| CXCL2 | GROβ, SCYB2, GRO2, MIP-2α, MGSA-β |
| CXCL3 | GROγ, MIP-2β |
| CXCL4 | PF-4 |
| CXCL5 | ENA-78, SCYB5 |
| CXCL7 | NAP-2 |
| CXCL9 | MIG |
| CXCL10 | IP-10 |
| CXCL11 | I-TAC |
| CXCL12 | SDF-1α, SDF-1β, SDF-1γ, SCYB12, PBSF, TLSF-α, TLSF-β, TPAR1 |
| CXCL13 | BCA-1 |
| CXCL14 | BRAK |
| CXCL15 |  |
| CXCL16 | SR-PSOX, SCYB16 |
| XCL1 | lymphotactin |
| CX3CL1 | fractalkine |
| GM-CSF |  |
| TLR2 |  |
| TLR4 |  |
| TLR9 |  |
| TRAIL | TNFSF10 |
| CD27L | TNFSF7, CD70 |
| STAT3 |  |
| MyD88 | - |
| iNOS | NOS type II, HEP-NOS |
| Receptors |  |
| IL-1R1 |  |
| IL-1R2 |  |
| IL-1RAP |  |
| IL-18R1 |  |
| IL-18RAP | IL-1R7, IL-18R2 |
| IL-18BP |  |
| IL-2Rb |  |
| IL-2Rg |  |
| IL-15Ra |  |
| gp130 |  |
| IL-6R | IL-6R-alpha, IL-6R 1, CD126 antigen |
| OSMR |  |
| IL-27R | WSX-1, Type I T-cell cytokine receptor, TCCR, CRL1 protein |
| IL-10R1 | IL-10Ra |
| IL-10R2 | IL-10Rb |
| IL-22RA1 |  |
| IL-12Rb1 | IL- 12RB1 |
| IL-12Rb2 | IL- 12RB2 |
| IL-23R |  |
| IL-17R | IL-17AR |
| CCR1 | CMKBR1, CC-CKR1, HM145, MIP-1a-R, RANTES-R, LD78-R |
| CCR2 | CMKBR2, CC-CKR2, CCR2A, CCR2B, MCP-1-R, mJE-R |
| CCR3 | CMKBR3, CC-CKR3, CC-CKR1-like 2, MIP-1α-R-like 2, eotaxin-receptor |
| CCR4 | CMKBR4, CC-CKR-4, K5-5 |
| CCR5 | CMKBR5, CC-CKR5, ChemR13, CD195, HIV-1 fusion coreceptor |
| CCR6 | CMKBR6, STRL22, GPR29, GPR-CY4, CKR-L3, DRY6, LARC-R |
| CCR7 | CMKBR7, BLR2, EBI1, CDw197, AMG1 |
| CCR8 | CMKBR8, CMKBRL2, CKR-L1, TER1, GPR-CY6, ChemR |
| CCR9 | CMKBR9, GPR-9-6, CC-CKR-9, CCR9A, CCR9B |
| CCR10 | CCR9, CCR10A, CCR10B |
| CCR11 | CC CKR-like 2, HCR, CRAM-A, CRAM-B, CKRX, E01, L-CCR |
| CXCR1 | IL8RA |
| CXCR2 | IL8RB |
| CXCR3 |  |
| CXCR4 |  |
| CXCR5 | BLR1 |
| CXCR6 | TYMSTR, STRL33, BONZO |
| XCR1 |  |
| CX3CR1 |  |
